# Mutations linked to loss of cell cycle control can render cells responsive to local differentiation cues

**DOI:** 10.17912/micropub.biology.000481

**Published:** 2021-10-01

**Authors:** Kara L Cerveny, Hannah Bronstein, Olivia Hagen, Dayna B Lamb, Grace Martin, Ingrid Tower, Avery Van Duzer, Evan Welch, Máté Varga

**Affiliations:** 1 Reed College; 2 Department of Genetics, ELTE Eötvös Loránd University, Budapest, Hungary

## Abstract

Cell behaviors such as survival, proliferation, and death are governed by a multitude of cues, both intrinsic and extrinsic. To test whether a wild-type environment could encourage the survival and/or differentiation of neuronal progenitor cells with impaired cell cycle progression, we transplanted cells from *cdk1, dtl, slbp, fbxo5, ahctf1, gins2, hdac1, mcm5, ssrp1a, *and* rbbp6 *mutant zebrafish embryos into wild-type embryos, creating chimeric zebrafish with mutant cells in the developing eye. We found that when cells from *cdk1, dtl, slbp, gins2, mcm5, *or* rbbp6* mutants were transplanted into wild-type hosts, survival and/or differentiation was almost always compromised in a manner consistent with cell-autonomous cell death. Interestingly, we observed that *fbxo5, ahctf1, hdac1, *or* ssrp1a* mutant cells survived and sometimes exhibited signs of differentiation when grafted into wild-type eyes.

**Figure 1. Survival and/or differentiation of ahctf1, ssrp1a, and fbxo5 mutant cells can be altered by a wild-type developing retinal environment:**
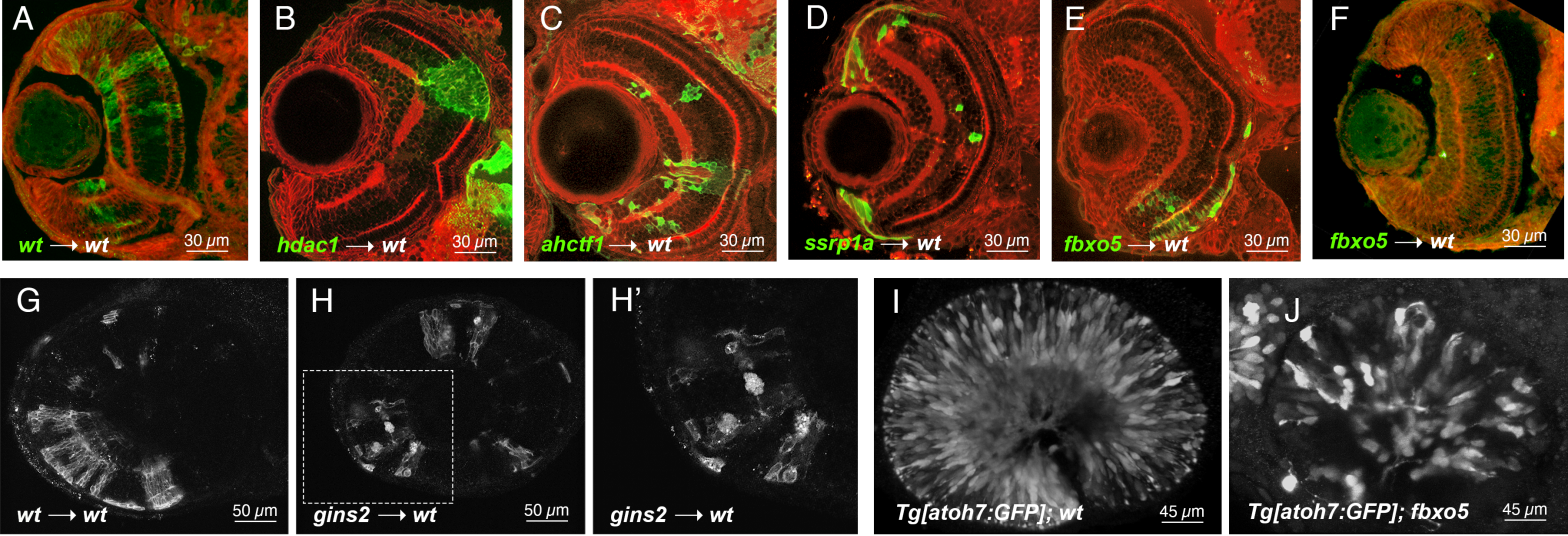
**A-F** Representative images of frontal cryosections of 3 dpf chimeric wild-type (wt) retinae containing GFP-labelled wild-type (A), *hdac1* mutant (B), *ahctf1* mutant (C), *ssrp1a* mutant (D) or *fbxo5* mutant (E-F) cells immunostained for GFP (green cells from donor embryo) and beta-catenin (red cell boundaries and plexiform layers). **G-H** Lateral views of single 2µm-thick z-plane of whole-mount 3 dpf chimeric wild-type retinae containing wild-type (G) or *gins2* morphant (H) cells labeled with membrane-targeted RFP. H’ shows 3x enlargement of boxed region in H. **I-J** Lateral maximum intensity projection of ~50 hpf retinae from *Tg[atoh7:GFP]* embryos showing neurogenic gene expression in wild-type (I) or *fbxo5* mutant (J) retinae.

## Description

Cell behaviors such as survival, proliferation, and death are governed by a bevy of cues, both from within the cells themselves and from the local tissue environment. To test whether a wild-type environment could encourage the survival and/or differentiation of neuronal progenitor cells with impaired cell cycle progression, we created chimeric zebrafish embryos containing mutant cells in wild-type retinae. We transplanted 10-20 cells from blastula stage donors into the region of early gastrula stage wild-type hosts fated to be eye field. Donor and host embryo pairs were cultured together; donors were genotyped when possible at 1 day post fertilization (dpf). We screened all transplants at 1 dpf for survival and location of clones. Hosts that contained labeled clones in their eyes were fixed at 3 dpf and either cryosectioned before immunostaining or subjected to immunostaining as wholemounts. All embryos analyzed in this study were imaged with epifluorescence and/or confocal microscopy. Consistent with previous reports, we found that *cdk1, dtl, slbp, fbxo5, ahctf1, gins2, hdac1, mcm5, ssrp1a,* and *rbbp6* mutant retinae contained dying cells with pyknotic nuclei throughout the developing retinal neuroepithelium at 3 dpf. Moreover, all of these mutant embryos had eyes that were noticeably smaller than their wild-type siblings (Table 1; references therein). When cells from mutant embryos were transplanted into wild-type hosts, survival and/or differentiation was almost always compromised in a manner consistent with cell-autonomous cell death. In particular, wild-type hosts that contained clones of *cdk1, dtl, mcm5,* and *rbbp6* mutant cells at 1 dpf rarely contained visible clones by 3 dpf (see Table 1 for numbers of chimeras analyzed and how many clones survived until 3 dpf ). For those clones that were visible, they were small (e.g., compare Fig 1A and 1F) and/or exhibited signs of apoptosis (e.g. Fig 1H). For example, by transplanting *gins2* morphant cells labeled with a membrane-targeted red fluorescent protein into wild-type embryos, we observed mutant cells blebbing and/or fragmenting when integrated into wild-type retinae (Fig 1H) whereas wild-type sibling cells integrated fully into the host environment, highlighting typical retinal neuronal morphologies (Fig 1G).

Clear evidence of mutant cell survival and/or differentiation was found in chimeric retinae containing *ahctf1, ssrp1a*, or *fbxo5* homozygous mutant cells in wild-type host retinae (Fig. 1C-E, Table 1). As we previously reported, we confirmed that *ahctf1* mutant cells transplanted into wild-type eyes appeared to survive and differentiate (Fig 1C, n = 19 transplants; (Cerveny *et al.* 2010)). The protein encoded by the *ahctf1* gene (also known as *elys*) has been implicated in a number of cell-cycle related functions including kinetochore assembly and nuclear pore assembly (e.g., (Rasala *et al.* 2006)). We reasoned that mutations in other ancillary cell-cycle proteins may also be susceptible to survival and/or differentiation cues in the wild-type retinal environment. To test this hypothesis, we examined the effects of a wild-type retinal environment on *ssrp1a* mutant cells. The *ssrp1a* gene encodes a component of the conserved facilitates chromatin transcription (FACT) complex and has been implicated in cell cycle control at the level of nucleosome remodeling necessary for DNA transcription, DNA replication, and DNA repair (Liu *et al.* 2020). Interestingly, we observed that *ssrp1a* mutant cells transplanted into wild-type embryos were rarely found in the differentiated retina and almost always in the ciliary marginal zone (CMZ), a region of the retina that contains a source of stem and progenitor cells throughout the life (Fig 1D, n=10 transplants).

The *fbxo5* gene encodes a protein that is both a substrate for and inhibitor of anaphase-promoting complex/cyclosome (APC/C), regulating the re-replication block, an essential step in the cell cycle (Cappell *et al.* 2018). Previous studies in zebrafish indicated that *fbxo5* can function both cell-autonomously and non-autonomously (Riley *et al.* 2010) and that *fbxo5* regulates genomic integrity and proliferation (Rhodes *et al.* 2009). We observed cell survival and differentiation in approximately one-third of *fbxo5*-wild-type chimeras. For instance, *fbxo5* mutant cells survived and appeared to differentiate in 10/34 transplants (Fig 1E). Interestingly, the majority (8/10) of chimeric retinae with surviving and differentiating *fbxo5* mutant cells were located in the ventral retina. In the remaining approximately two-thirds of our sample, however, *fbxo5* mutant clones appeared to be lost by cell death and/or not differentiate in wild-type environments (Fig 1F). These data suggest that these mutant cells might be especially sensitive to slight differences in age of the donor embryo at time of transplantation, differences in location of transplanted cells, or stochastic fluctuations in gene expression (e.g.,(Trimarchi *et al.* 2008)) in the transplanted cells or host embryos. Because multiple people performed these transplants, it is also possible that some of the variability we observe is due to batch effects and individual technique.

Previous reports have shown that zebrafish embryos carrying homozygous mutations in *fbxo5* (also known as *emi1)* still exhibit some neuronal differentiation (Zhang *et al.* 2008; Riley *et al.* 2010). We found that a small, but notable fraction of *fbxo5* mutant retinal progenitor cells still express the neurogenic gene *atoh7*, as observed with the *atoh7:GFP* transgene (Poggi *et al.* 2005) and form some retinal ganglion cells (compare Fig 1I-J). The same has been shown for *ahctf1* mutants (Davuluri *et al.* 2008; Cerveny *et al.* 2010) and *ssrp1a* mutants (Koltowska *et al.* 2013). It is possible, therefore, that the effects of the wild-type environment on *fbxo5*, *ahctf1* , or *ssrp1a* deficient cells result from stochastic expression of some early neurogenic genes that prime cells for survival and/or differentiation in the neural retina.

Finally, our transplant studies also confirmed previous reports that mutations in histone deacetylase 1, *hdac1,* are linked to cell autonomous hyperproliferation in the retina (Stadler *et al.* 2005; Yamaguchi *et al.* 2005, Fig 1B). When we examined *hdac1* mutant cells that had integrated into wild-type chimeric retinae at 3 dpf, a point at which apoptotic cells are found scattered throughout the *hdac1* mutant retinae (Yamaguchi *et al.*, 2005), we did not observe pyknotic nuclei or cell blebbing, two key hallmarks of apoptosis. Instead, we observed large clones that interrupted retinal lamination and did not exhibit neuronal morphologies (e.g., Fig 1B). This finding raises the possibility that a wild-type retinal environment supports the survival of these proliferative cells but does not promote their cell cycle exit and/or differentiation.

The difference in susceptibility of mutant cells to the wild-type environment may be explained, in part, by the distinct functions of the mutated genes. Of note, the only mutant cells that significantly survived and/or differentiated in a wild-type environment (*ahctf1*, *ssrp1a, and hdac1)* carry mutations in genes that impact cell cycle progression but are not part of the canonical cell cycle machinery. We speculate that mutations in genes that are not directly linked to the cell cycle but nonetheless exhibit cell cycle defects may be part of a redundant regulatory network and therefore are more likely to respond to survival and differentiation factors in a wild-type environment.

## Methods

**Zebrafish lines**

Eggs were collected by natural spawning, raised at either 25˚C or 28.5˚C in E3 embryo medium (Nüsslein-Volhard, C. and Dahm, R. 2002) and staged according to Kimmel *et al.*, 1995. After gastrulation and before 24 hours post-fertilization, embryos were cultured in 0.003% phenylthiourea (PTU, Sigma) in E3 to prevent pigment formation. Lines used in this study and associated references are listed in Table 1. Adult zebrafish were cared for with protocols approved by the Reed College IACUC.

**Cell transplants**

Similar to previously published studies (e.g., (Cerveny *et al.* 2010; Turner *et al.* 2019), donor embryos were injected at the 1-cell stage with ~20 ng of GFP mRNA synthesized from linearized pCS2-GFP or membrane-targeted RFP mRNA synthesized from linearized pCS2-membrane-targeted mCherry with the T7 mMessage mMachine kit (Ambion) according to manufacturer’s instructions. Host and donor embryos were grown at 28.5˚C until sphere stage (approximately 4 hours post-fertilization) and then 10-20 fluorescently labelled cells were removed from donor embryos and transplanted into the animal pole of unlabeled host embryos. Donor and host embryos were incubated overnight at 28.5˚C. All embryos were screened and E3 was exchanged for PTU in E3. Donors were identified by visual inspection and by PCR and restriction digest mediated genotyping. Genotyping protocols for each line can be found at Zebrafish International Resource Center (ZIRC; http://zebrafish.org/home/guide.php) and in relevant references (see Reagents; Table 1). For *gins2* experiments, 1-cell stage embryos were first injected with ~1 nl of 1 mM *gins2* morpholino (Gene Tools, Philomath, OR; 5’-GGGGTGAGTCAATTTATAATCTAC-3’), a dose that phenocopies *gins2*^-/-^ mutants (Varga *et al.* 2020) and then injected with ~10 ng of membrane-targeted RFP mRNA

**Immunohistochemistry, imaging, and analysis**

After fixation, wholemount embryos were either subjected to immunohistochemistry as previously described (Cerveny *et al.* 2010) or were cryoprotected in 15% and then 30% sucrose before being embedded in Optimal Cutting Temperature (OCT) resin and cut into 30 µm thick sections that were collected on charged glass slides (Polysciences, cat number: 24216) and stained with the following antibodies: beta-catenin (mouse, 1:250 dilution; Sigma, C7207); GFP (chicken, 1:250 dilution, Abcam, ab139709); RFP (rabbit, 1:500 dilution, MBL, PM005). Nuclei were counterstained with DAPI (1 µg/ml from a 1 mg/ml stock in DMSO; Sigma) or sytox orange (1:10,000 dilution, Invitrogen). All images pictured were captured on a Nikon A1+ confocal with a long working distance 25X, 1.1 NA water immersion lens or a Leica SP8 confocal with a 20x 0.8NA water immersion lens.

## Reagents

**Table:** 

**Table 1.** Cell cycle mutants examined for responsiveness to a wild-type environment by chimeric analysis in zebrafish retinae.				
Mutant	Molecular function of mutated gene according to literature	Phenotype linked to cell cycle defect as reported in literature	Phenotypes of mutant cells when transplanted into WT retinae as examined by cell morphology	References
*cdk1^hi3235Tg^*	binds various cyclins promoting entry into S-phase and mitosis	stall in G1, G1/S, S phases, apoptosis	apoptosis (12 chimeras analyzed; only 5/12 chimeras contained small clones (1-3 cells) by 3 dpf)	(Amsterdam *et al.* 2004); this study
*ssrp1a^s819^*	component of FACT complex, remodels chromatin, functions during transcription, DNA replication and repair	arrest in S phase, apoptosis	survival in the CMZ and RPE but some quiescence and apoptosis in neural retina (10 chimeras analyzed; 10/10 chimeras contained clones by 3 dpf)	(Koltowska *et al.* 2013); this study
*dtl^hi3627Tg^*	E3-ubiquitin ligase, regulates cyclin-dependent kinase inhibitors	arrest in late S/early G2, apoptosis	apoptosis (18 chimeras analyzed; only 3/18 chimeras contained very small clones (1-3 cells) by 3 dpf)	(Sansam *et al.* 2010); this study
*slbp1^ty77e^*	binds stem-loop structure of histone mRNAs, stabilizes pre-mRNA-snRNP interactions	stall in G1/S, apoptosis	apoptosis (17 chimeras analyzed; 16/17 chimeras contained visible clones by 3 dpf)	(Turner *et al.* 2019); this study
*fbxo5^hi2648Tg^*	APC/C inhibitor, known to block re-replication	primarily arrest in G2/M, apoptosis.	some differentiation but also some apoptosis; highly variable (34 chimeras analyzed; 10/34 chimeras had visible clones by 3 dpf; 24/34 chimeras had very small (1-3 cells), but visible clones by 3 dpf)	(Rhodes *et al.* 2009; Riley *et al.* 2010; Zhang *et al.* 2008); this study
*ahctf1^ti262c^*	kinetochore protein also required for nuclear pore assembly	cycle slowly, stalling in either G1/S or G2/M	survival and differentiation (19 chimeras analyzed; 19/19 chimeras had visible clones by 3 dpf)	(Cerveny *et al.* 2010; Davuluri *et al.* 2008); this study
*gins2^u773^*(also used morpholinos)	DNA replication initiation and progression	Delayed/prolonged S phase, apoptosis	apoptosis (26 chimeras analyzed; 26/26 chimeras had visible clones by 3 dpf)	(Varga *et al.* 2020); this study
*hdac1^hi1618Tg^*	removes acetyl groups linked to lysine residues typically found on histones	unable to exit the cell cycle; slowly proliferate and do not differentiate	survival and proliferation (14 chimeras analyzed at 4 dpf; 9 chimeras analyzed at ~3 dpf; all contained visible clones)	(Yamaguchi *et al.* 2005; Zhou *et al.* 2011); this study
*mcm5^m850^*	component of a DNA helicase, required during S-phase	prolonged S phase, apoptosis	apoptosis (15 chimeras analyzed; only 10/15 chimeras contained very small clones (1-3 cells) by 3 dpf)	(Ryu *et al.* 2005); this study
*rbbp6^hi2993Tg^*	E3-ubiquitin ligase with functions linked to DNA replication and DNA repair	predicted to arrest in G1/S, apoptosis	apoptosis (12 chimeras analyzed; only 1/12 chimeras contained very small clones (1-3 cells) by 3 dpf)	(Amsterdam *et al.* 2004); this study
*Tg[atoh7:GFP]^rw021Tg^*	labels progenitors as they are being specified as retinal ganglion cells			(Poggi *et al.* 2005)

## References

[R1] Cappell SD, Mark KG, Garbett D, Pack LR, Rape M, Meyer T (2018). EMI1 switches from being a substrate to an inhibitor of APC/C^CDH1^ to start the cell cycle.. Nature.

[R2] Cerveny KL, Cavodeassi F, Turner KJ, de Jong-Curtain TA, Heath JK, Wilson SW (2010). The zebrafish flotte lotte mutant reveals that the local retinal environment promotes the differentiation of proliferating precursors emerging from their stem cell niche.. Development.

[R3] Davuluri G, Gong W, Yusuff S, Lorent K, Muthumani M, Dolan AC, Pack M (2008). Mutation of the zebrafish nucleoporin elys sensitizes tissue progenitors to replication stress.. PLoS Genet.

[R4] Kimmel CB, Ballard WW, Kimmel SR, Ullmann B, Schilling TF (1995). Stages of embryonic development of the zebrafish.. Dev Dyn.

[R5] Koltowska K, Apitz H, Stamataki D, Hirst EM, Verkade H, Salecker I, Ober EA (2013). Ssrp1a controls organogenesis by promoting cell cycle progression and RNA synthesis.. Development.

[R6] Liu Y, Zhou K, Zhang N, Wei H, Tan YZ, Zhang Z, Carragher B, Potter CS, D'Arcy S, Luger K (2019). FACT caught in the act of manipulating the nucleosome.. Nature.

[R7] Nüsslein-Volhard C, Dahm R. 2002 <i>Zebrafish: A practical approach</i>. Oxford University Press, Oxford, UK.

[R8] Poggi L, Vitorino M, Masai I, Harris WA (2005). Influences on neural lineage and mode of division in the zebrafish retina in vivo.. J Cell Biol.

[R9] Rasala BA, Orjalo AV, Shen Z, Briggs S, Forbes DJ (2006). ELYS is a dual nucleoporin/kinetochore protein required for nuclear pore assembly and proper cell division.. Proc Natl Acad Sci U S A.

[R10] Rhodes J, Amsterdam A, Sanda T, Moreau LA, McKenna K, Heinrichs S, Ganem NJ, Ho KW, Neuberg DS, Johnston A, Ahn Y, Kutok JL, Hromas R, Wray J, Lee C, Murphy C, Radtke I, Downing JR, Fleming MD, MacConaill LE, Amatruda JF, Gutierrez A, Galinsky I, Stone RM, Ross EA, Pellman DS, Kanki JP, Look AT (2009). Emi1 maintains genomic integrity during zebrafish embryogenesis and cooperates with p53 in tumor suppression.. Mol Cell Biol.

[R11] Riley BB, Sweet EM, Heck R, Evans A, McFarland KN, Warga RM, Kane DA (2010). Characterization of harpy/Rca1/emi1 mutants: patterning in the absence of cell division.. Dev Dyn.

[R12] Stadler JA, Shkumatava A, Norton WH, Rau MJ, Geisler R, Fischer S, Neumann CJ (2005). Histone deacetylase 1 is required for cell cycle exit and differentiation in the zebrafish retina.. Dev Dyn.

[R13] Trimarchi JM, Stadler MB, Cepko CL (2008). Individual retinal progenitor cells display extensive heterogeneity of gene expression.. PLoS One.

[R14] Turner KJ, Hoyle J, Valdivia LE, Cerveny KL, Hart W, Mangoli M, Geisler R, Rees M, Houart C, Poole RJ, Wilson SW, Gestri G (2019). Abrogation of Stem Loop Binding Protein (Slbp) function leads to a failure of cells to transition from proliferation to differentiation, retinal coloboma and midline axon guidance deficits.. PLoS One.

[R15] Varga M, Csályi K, Bertyák I, Menyhárd DK, Poole RJ, Cerveny KL, Kövesdi D, Barátki B, Rouse H, Vad Z, Hawkins TA, Stickney HL, Cavodeassi F, Schwarz Q, Young RM, Wilson SW (2020). Tissue-Specific Requirement for the GINS Complex During Zebrafish Development.. Front Cell Dev Biol.

[R16] Yamaguchi M, Tonou-Fujimori N, Komori A, Maeda R, Nojima Y, Li H, Okamoto H, Masai I (2005). Histone deacetylase 1 regulates retinal neurogenesis in zebrafish by suppressing Wnt and Notch signaling pathways.. Development.

[R17] Zhang L, Kendrick C, Jülich D, Holley SA (2008). Cell cycle progression is required for zebrafish somite morphogenesis but not segmentation clock function.. Development.

